# Assessing InSAR observability of landslides interfering with bridges

**DOI:** 10.1038/s41598-026-41011-6

**Published:** 2026-03-02

**Authors:** Erica Cernuto, Diana Salciarini, Filippo Ubertini, Giorgia Giardina

**Affiliations:** 1 Department of Civil and Environmental Engineering, University of Perugia, Via Goffredo Duranti, 93, 06126 Perugia, Italy; 2 Department of Geoscience and Engineering, Delft University of Technology, Stevinweg 1, 2628 CN Delft, The Netherlands

**Keywords:** InSAR, Coverage index, Geomorphological factors, Landslide–bridge interaction, Deformation velocity distribution, Engineering, Environmental sciences, Natural hazards, Solid Earth sciences

## Abstract

Landslides are among the most widespread natural hazards worldwide and a major cause of disruption to infrastructure networks, with significant impacts on safety and territorial resilience. Understanding the conditions under which they can be effectively monitored is crucial for reducing risk and supporting mitigation strategies. Satellite radar interferometry enables the detection of ground deformation with high precision and wide spatial coverage, but the main challenge lies in identifying when this technology can detect and characterise landslides, as radar visibility is strongly influenced by topography and acquisition geometry. This study addresses this challenge by analysing the interferometric observability of landslides near infrastructure, integrating European Ground Motion Service data with the Italian landslide inventory. This combination enables a systematic quantification of how geomorphological factors and movement characteristics influence the radar detectability of landslides interacting with infrastructure. The analysis shows how topographic settings and movement types control radar visibility, and how InSAR can identify activity states and reveal the internal variability of deformation. The comparison between landslides and interfering bridges highlights differences attributable to local conditions, emphasising the importance of interpreting structural deformation within the geomorphological context. The results provide a quantitative basis to guide monitoring strategies and risk management in complex infrastructural settings.

## Introduction

In recent years, the interaction between landslides and linear infrastructures, such as bridges, has attracted growing scientific interest, driven by the need to better understand the mechanisms through which ground movements affect structural response and integrity^1,2^. Several recent studies have highlighted that even slow or localised slope movements can induce significant deformations in structural elements, potentially compromising the overall stability of the structure^3–5^. The significance of these phenomena is amplified by the increasing global vulnerability of infrastructure. In Italy, statistical analyses of structural collapses indicate that approximately 73$\%$ of failures are associated with natural hazards, among which landslides and floods represent the predominant causes^6^. The impact of such hazards, particularly those of geomorphological origin, is especially relevant at the global scale: a recent worldwide assessment of geo-hazard risk (including landslides and subsidence) for long-span bridges revealed that over 20$\%$ of these structures fall within the very high-risk category, and more than 50$\%$ are classified as high-risk^7^. Landslide monitoring therefore represents a key component in risk management and infrastructure protection, enabling the timely detection of active deformations and the planning of effective mitigation measures. However, the widespread occurrence of landslides and their frequent location in morphologically complex terrain makes it challenging to ensure adequate coverage using conventional techniques alone. This highlights the need for observational tools capable of operating on a large scale, continuously, and with sufficient spatial resolution to capture the distribution of ground deformation.

Interferometric Synthetic Aperture Radar (InSAR) has emerged as a particularly promising technique in this context^8,9^, offering the ability to measure ground displacements in a non-invasive manner, regardless of lighting or weather conditions, and over wide areas with millimetre-level precision^10,11^. These features make radar interferometry especially effective in contexts where landslides potentially interact with infrastructure^12–15^, as even minimal ground movements can provide early indications of evolving instability and support the assessment of soil–structure interaction. The development of advanced differential interferometry techniques has significantly enhanced the potential of radar remote sensing to detect ground deformation. In particular, Multi Temporal InSAR (MT-InSAR) enables the identification and long-term monitoring of specific radar targets, known as Persistent Scatterers (PS), which maintain a stable electromagnetic response over time^16^. These targets typically correspond to well-reflecting natural or man-made features, such as rock outcrops or isolated structures, and allow the detection of surface displacements even in morphologically complex environments^16,17^. These techniques have been successfully applied to landslide monitoring in numerous case studies reported in the literature^18–21^.

In recent years, the availability of InSAR data from Sentinel-1 missions of the Copernicus programme, along with the development of operational services at the continental scale, such as the European Ground Motion Service (EGMS)^22–25^, has opened new perspectives for systematic monitoring of surface deformation^26^. The free accessibility and regular coverage of these data has encouraged the widespread adoption of large-scale landslide monitoring approaches, as demonstrated by numerous recent scientific contributions^27–29^. However, the practical applicability of InSAR remains strongly constrained by geomorphological, environmental, and geometric factors that affect radar visibility and measurement quality^10^. Among these, perspective distortions related to the angle of incidence of the radar signal with respect to slope geometry are particularly significant, potentially causing layover or shadowing effects that reduce interferometric coverage in large areas^30^. In many cases, potentially active landslides are only partially or entirely not covered by radar data, due to dense vegetation, complex soil morphology, or unfavourable slope orientations relative to the satellite line of sight, representing one of the main limitations of the technique^31^. To systematically assess the effectiveness of interferometric observation when applied to landslide phenomena, it is essential to rely on a robust and structured knowledge base regarding the distribution and characteristics of landslides. In this regard, the integration of geological inventories and satellite-based measurements represents a crucial step.

Several studies have already demonstrated the value of integrating InSAR products with landslide inventories to improve mapping, characterisation, and updating of slope instabilities at various scales. For instance, at the regional scale in Italy, Rosi et al. (2018)^32^ updated the Tuscany landslide inventory using persistent scatterer interferometry data combined with geomorphological features, highlighting the potential of remote sensing for inventory enrichment. Similarly, Confuorto et al. (2023)^33^ exploited satellite measurements to verify and refine landslide catalogues at regional level, identifying unmapped or reactivated phenomena. Finally, Medici et al. (2025)^34^ integrated EGMS data with landslide inventories in Great Britain to identify active deformation areas and highlight unmapped moving landslides for closer inspections. Extending these approaches to the national level in Italy requires a comprehensive landslide inventory such as the Inventory of Landslide Phenomena in Italy (IFFI), coordinated by the Italian Institute for Environmental Protection and Research (ISPRA) in collaboration with Regional and Autonomous Provincial Authorities, constitutes the main national reference^35^. The inventory currently records over 620,000 landslides across the entire Italian territory, providing detailed information on their location, extent, type of movement, and activity status^36^. This database is unique in the European context, offering a systematic and georeferenced representation of landslide occurrences, and serves as a foundational resource for territorial assessments, comparative analyses, and integration with satellite observations. The integration of the IFFI inventory with interferometric data offers a valuable opportunity to systematically investigate the radar detectability of landslides at a regional scale. Building on this information base, it becomes possible to explore the extent to which geomorphological and typological characteristics of the phenomena influence SAR coverage, as well as to assess the capability of InSAR to detect deformations potentially interacting with existing infrastructure.

This study proposes a quantitative framework to assess the interferometric observability of landslides interacting with bridges at regional scale, integrating EGMS data with the official Italian landslide inventory and geomorphological information. The main objective is to identify the conditions that influence radar coverage of landslides, by jointly considering the type of movement, the morphological characteristics of the slope, and the orbital geometry of the data (ascending and descending). The analysis focuses on two distinct regions of the Italian territory, where landslides potentially interacting with bridges were selected, to further investigate the effectiveness of InSAR in detecting potentially critical deformations near vulnerable structural elements. For landslides that exhibit good interferometric coverage, the internal variability of deformation is also analysed, by evaluating the distribution of displacement velocities and comparing the signals detected on the bridges with those observed in the surrounding unstable areas. The results of this study advance understanding of radar detectability of landslides-infrastructure settings, provide useful insights for preliminary vulnerability assessments and support the operational integration of InSAR data into decision-making processes.

## Investigated areas

**Fig. 1:**
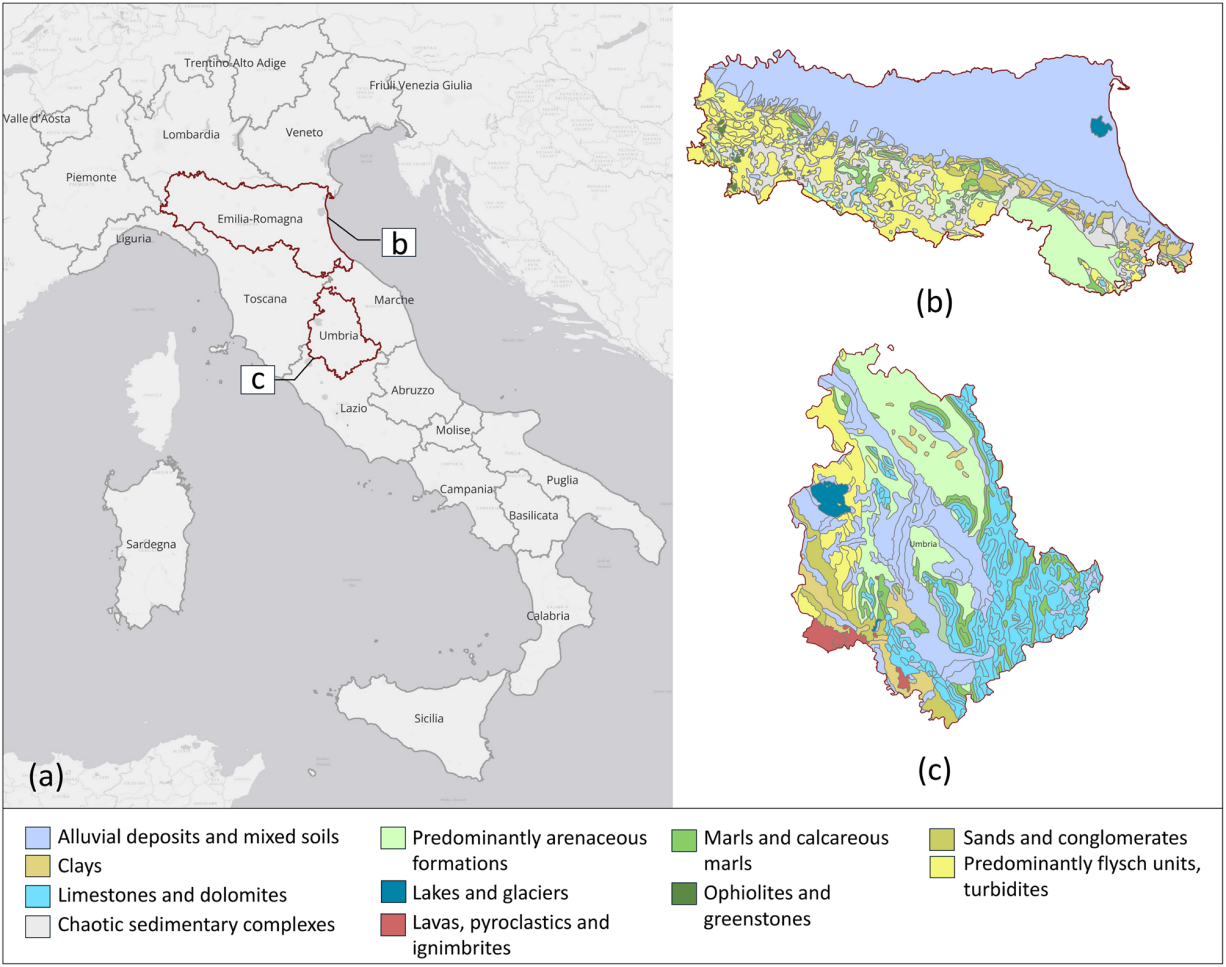
Location of the two study areas within Italy (**a**) and corresponding geolithological maps of Emilia-Romagna (**b**) and Umbria (**c**) at 1:500,000 scale. Lithological units follow the official dataset classification. Data were accessed via the Web Map Service (WMS) of the Italian National Geoportal (https://gn.mase.gov.it/portale/servizio-di-consultazione-wms), and the maps were composed and visualised using QGIS v3.42.0 (https://qgis.org).

The analysis was carried out in two regions of the Italian Apennines, Emilia-Romagna and Umbria (Fig. 1a), selected as representative examples of morphologically complex settings with a high density of both landslides and linear infrastructures. The selection was based on the availability of consistent and comparable regional-scale datasets, as well as the opportunity to test the proposed procedure in areas characterised by different geological and infrastructural conditions. Although both regions belong to the Apennine structural domain, they exhibit marked differences in the spatial distribution of slope instabilities, the density of infrastructure networks, and lithostratigraphic characteristics. This diversity offers an ideal framework for assessing how SAR coverage and the interferometric visibility of landslides vary according to geological and infrastructural factors, and for analysing potential interactions between slope movements and linear assets.

**Fig. 2:**
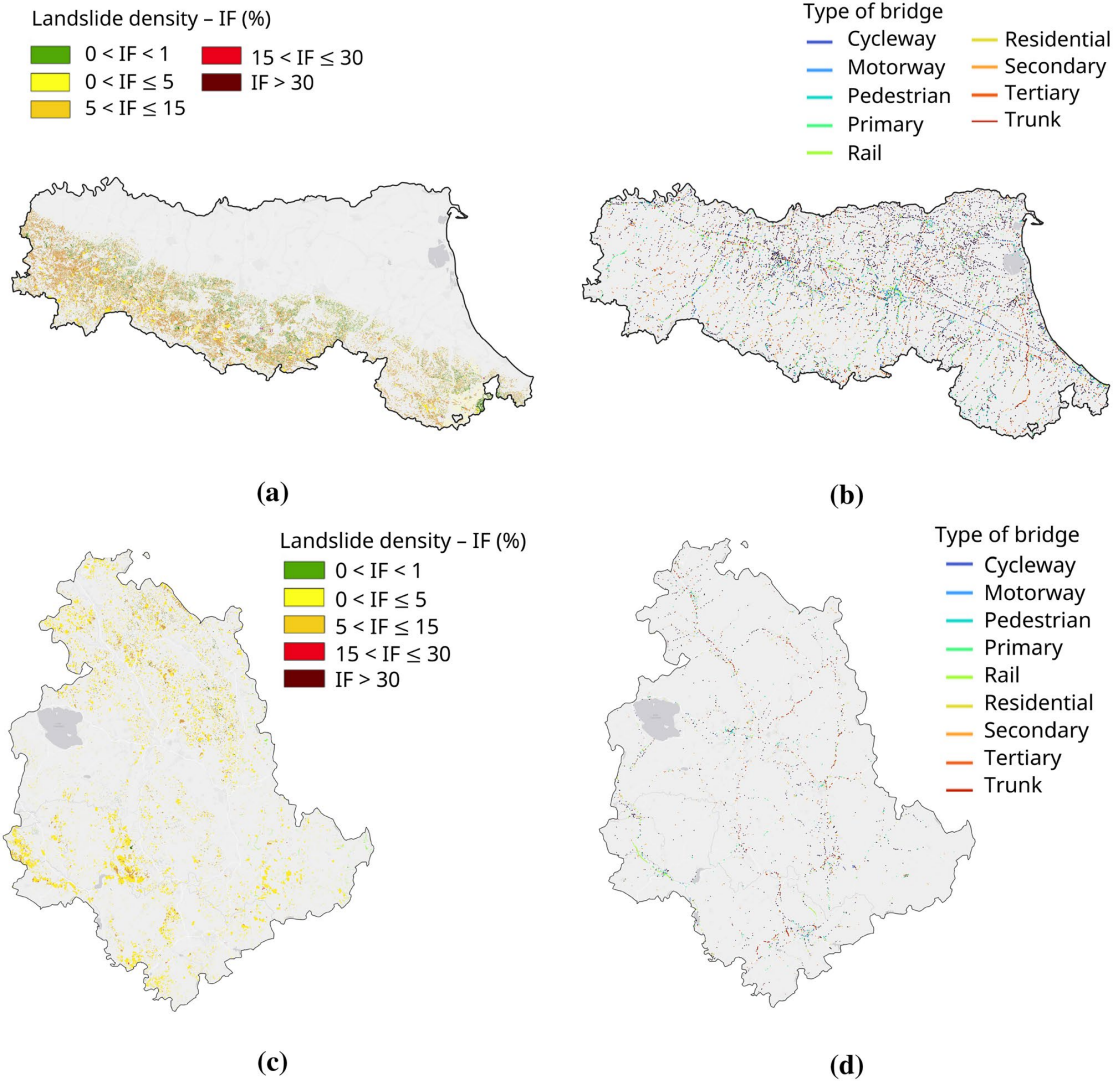
Emilia-Romagna and Umbria regions showing: (**a–c**) landslide density (ratio between landslide area and total area) based on the IFFI (Italian Landslide Inventory), with data accessed from the ISPRA open-data portal (https://idrogeo.isprambiente.it/app/page/open-data); (**b–d**) distribution and classification of bridges extracted from OpenStreetMap (OSM). Maps were generated and visualised using QGIS v3.42.0 (https://qgis.org).

From a geological and morphological perspective, Emilia-Romagna features a predominantly hilly and mountainous landscape, dominated by clay and marly-arenaceous sequences that confer an intrinsic predisposition to slope instability, both shallow and deep-seated^37^ (Fig. 1b). According to IFFI^35^, a total of 80,259 landslides have been recorded in Emilia-Romagna, mostly concentrated along the Apennine foothills, with the highest density in the south-western part of the region. In contrast, the north-eastern plain is virtually unaffected by significant slope instabilities (Fig. 2a). This distribution reflects the combined influence of the lithological and morphological characteristics of the region: the Apennine slopes, predominantly composed of weak lithotypes such as clays, marls, and flysch and characterized by moderate to steep gradients, are naturally prone to landslide processes. Conversely, the flat areas of the north-eastern plain, formed by recent alluvial deposits and characterised by near-zero slopes, are intrinsically less susceptible to such phenomena. The most frequent types of landslides include rotational and translational slides, slow earth flows and complex failures, whereas landslide phenomena such as rockfalls, rapid debris flows and lateral spreads occur only sporadically.

Umbria, on the other hand, exhibits a more heterogeneous morphology, characterised by alternating calcareous and flysch ridges and intermontane basins. The presence of marly–arenaceous formations and lithologies with lower mechanical strength mainly promotes slow and diffuse landslides, while the carbonate reliefs are affected by localised rockfalls along steep rock faces^38^ (Fig. 1c). The spatial distribution of slope failures in Umbria is more homogeneous than in Emilia-Romagna, with no clear concentration in any specific morphological zone (Fig. 2c). This pattern reflects the variability in geological and geomorphological conditions across the region. According to the IFFI inventory^35^, 31,540 landslides are recorded in the region, mainly consisting of slides, flows and complex failures, with sporadic episodes of rockfall.

Given the widespread occurrence of landslides in the two regions under analysis and their potential impact on linear infrastructure, the study also considered the existing infrastructural network, with a focus on road and railway bridges, as well as minor crossings. The inventory of these structures was compiled using data from OpenStreetMap, through targeted queries performed within a GIS environment (QuickOSM), identifying a total of 13,845 bridges in Emilia-Romagna (Fig. 2b) and 3250 in Umbria (Fig. 2d). The coexistence of a high density of landslides and a widespread infrastructural network highlights the need for a systematic approach to investigate potential cases of interaction. This regional framework provides the reference setting for the subsequent analyses, which aim to explore the interferometric visibility of landslides and to evaluate the deformation behaviour of those occurring in the proximity of infrastructure.

## Datasets and method

**Fig. 3:**
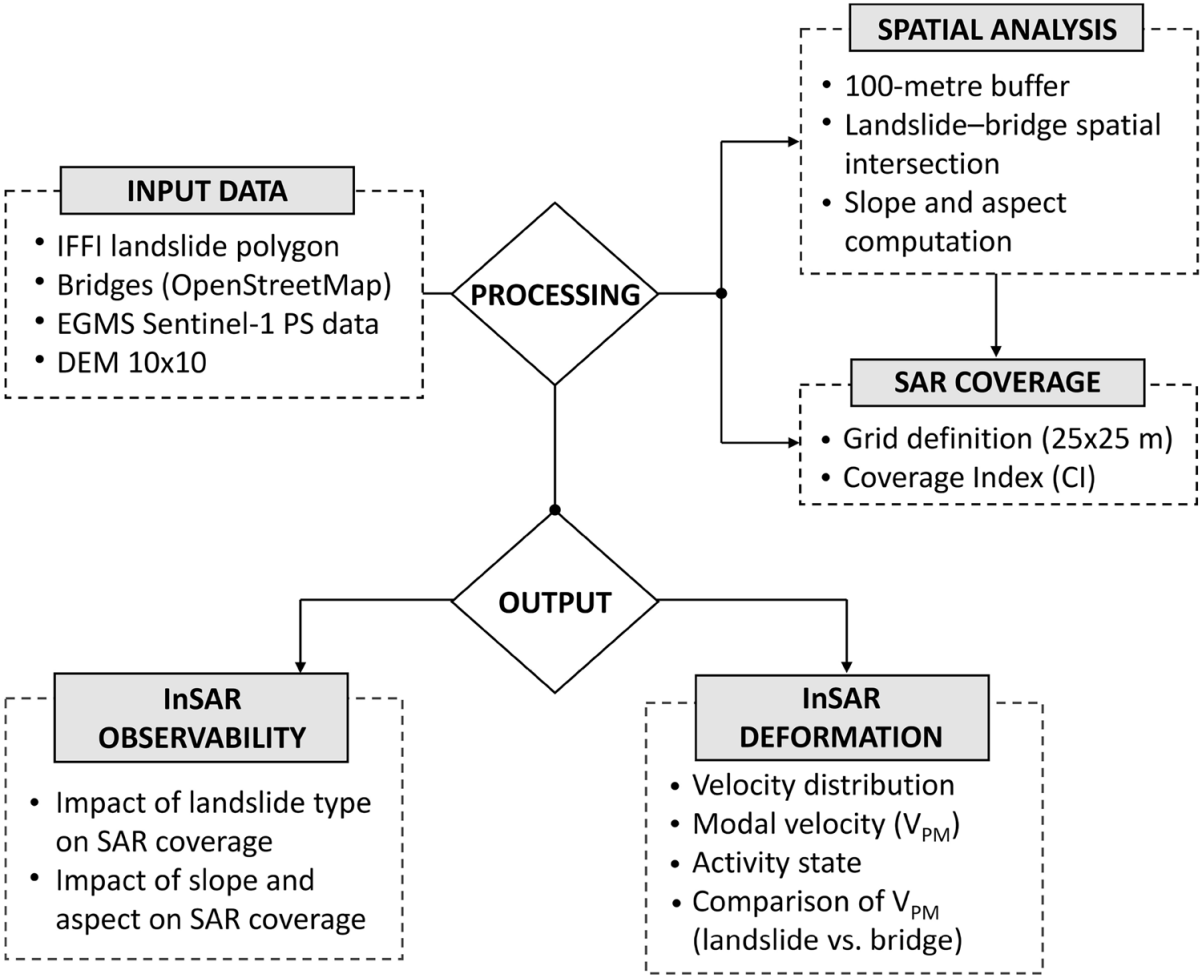
Flowchart of the adopted methodology.

The methodological approach proposed in this study integrates GIS-based spatial analysis, information from the IFFI inventory, and Sentinel-1 (EGMS) interferometric data to investigate the potential interaction between bridges and landslides. The workflow (Fig. 3) is structured into four main steps. In the first step, the landslides potentially interacting with the bridges were identified by defining a buffer around each structure and intersecting it with the landslide polygons provided by the IFFI inventory. In the second step, the interferometric coverage of these landslides was assessed using Sentinel-1 PS data through a grid-based analysis designed to quantify SAR visibility. The third step examined the influence of landslide type and geomorphological factors (slope and aspect) on SAR observability. Finally, for landslides with sufficient radar coverage, the LOS deformation velocity was analysed to assess their activity state and internal variability; the resulting velocities were then compared with those observed on the interacting bridges, with the aim of evaluating potential deformation correspondences between the landslide and the infrastructure.

### Datasets

The analysis was carried out within a Geographic Information System (GIS) environment using QGIS (Quantum Geographic Information System) and, to assess the actual risk of interaction between infrastructure and landslide phenomena, a 100-m buffer was defined around each bridge, considered representative of the area potentially affected by the direct or indirect effects of nearby slope failures. Since the IFFI inventory provides, for each landslide, a validated cartographic perimeter that represents its actual extent, the spatial intersection between these buffers and the mapped landslide areas made it possible to precisely identify both the potentially affected infrastructures and the slope failures effectively located near the bridges. This refined subset constituted the basis for the subsequent interferometric analysis, aimed at assessing the visibility of landslide phenomena and the spatial coverage offered by SAR data. InSAR data used in this study were derived from Sentinel-1 imagery, accessed through the EGMS service, which is part of the Copernicus program, managed by the European Environment Agency^25^. This open and freely accessible service provides point-based ground deformation measurements obtained from Sentinel-1 images, with a temporal resolution of six days^39^. In this context, Sentinel-1 C-band sensors provide a favourable balance between spatial resolution and vegetation penetration compared to X-band and L-band sensors, and their wide-swath acquisition capability supports applications at both regional and global scales^40,41^. Therefore, the use of C-band data within an openly accessible framework enables reproducible large-scale analyses and facilitates the application of the proposed methodology across different geographical contexts. This study used the “Calibrated” EGMS product, which provides average annual deformation velocities (mm/year) along the sensor Line of Sight (LOS), representing the one-dimensional projection of ground displacement between the satellite and the target. Two datasets were analysed, ascending (02/01/2019–25/12/2023) and descending (06/01/2019–29/12/2023), ensuring consistent temporal coverage. Although EGMS also provides a derived “Ortho” product with vertical and east–west velocity components resampled on a 100 m grid, this work focused on the original orbital geometries to directly assess radar coverage and PS distribution. This choice maintains control over visibility factors, such as slope morphology and orientation with respect to the LOS, and provides a consistent basis for future developments aimed at reconstructing the three-dimensional displacement components.

### Definition of SAR coverage

To quantify the interferometric coverage of landslide phenomena, each landslide polygon was divided into a regular grid of square cells, each measuring 25 m per side. This grid size was chosen as a compromise between two contrasting needs: on one hand, a grid that is too large may lead to an overly generalised representation, where a few interferometric points cover extensive portions of the landslide, resulting in a loss of spatial detail; on the other hand, an excessively fine grid increases the likelihood of cells containing no data, due to the limited density of PSs in the EGMS datasets. The analysis was carried out separately for each orbital geometry (ascending and descending). For each landslide, a Coverage Index (CI) was calculated, defined as the percentage of cells containing at least one PS relative to the total number of cells within the landslide polygon. Based on the CI value, landslides were classified into four coverage levels to ensure a consistent and operational interpretation of radar visibility: not covered (CI<25$\%$), where less than one quarter of the landslide area contains PS and information is insufficient for reliable assessment; poorly covered (25$\%\le$CI<50$\%$), allowing only partial interpretation; moderately covered (50$\%\le$CI<75$\%$), where PS are representative of most of the landslide area though some portions remain unobserved; and well covered (CI$\ge$75$\%$), in which at least three quarters of the landslide area include PS, enabling detailed and reliable deformation analyses. Although the threshold values are defined in a conventional manner, they were calibrated based on the actual distribution of PS in the analysed areas, the adopted spatial resolution (25×25 m), and the scale of the present study. Moreover, the choice of using four classes represents an effective compromise between informational detail and interpretability, facilitating comparisons between landslides and supporting the application of selection criteria in the subsequent stages of the analysis.

Unlike the visibility indices established in the literature, such as the R-index^42–44^ and the LU-index^42,43^ or the more recent CR-index^42^ and the $MPD\_m$ map^45^, which are primarily a priori analysis tools designed to assess the feasibility of InSAR monitoring by simulating geometric distortions and land cover influence, the CI proposed in this study represents an a posteriori measure of interferometric observability. While traditional indices are used to predict where measurement points (MP) might be detected, the CI quantifies the actual detectability using data already processed and validated by the EGMS service. In particular, although advanced models^44^ allow for the precise identification of critical areas such as far passive layover, topographically complex zones where the radar signal is distorted by the overlap of adjacent slopes, the CI provides an empirical validation of these limitations. It measures the extent to which a landslide surface is actually monitored for risk management purposes, recording as information gaps those effects of distortion and decorrelation that a priori models tend to theoretically predict. In this sense, the CI does not substitute predictive models but provides a systematic quantification of how geomorphological and environmental factors have actually conditioned the final distribution of PSs on a regional scale.

### Analysis of landslide types and geomorphological factors on InSAR observability

To investigate how landslide types affect interferometric observability, a cross-analysis was conducted between the classes defined in the IFFI inventory and the available SAR coverage level. The IFFI classification follows the criteria proposed by Varnes (1978)^46^ and updated by Cruden and Varnes (1996)^47^, which include landslide types such as translational or rotational slides, slow or rapid flows, complex landslides, topples and falls, as well as extensive areas affected by numerous rockfalls/topples, sinkholes, or shallow landslides. In addition, the category “n.d.” (not determined) is used in the IFFI inventory to indicate landslides whose typology cannot be identified with sufficient confidence. The purpose of the analysis is to assess whether, and to what extent, each landslide type exhibits different levels of observability, identifying potential patterns between landslide kinematics and interferometric coverage. This allows for a critical interpretation of the effectiveness of differential radar interferometry in detecting ground deformations associated with various landslide mechanisms, highlighting those types that are generally well monitored compared to others that present greater observational limitations. To this end, the percentage of landslides belonging to each interferometric coverage category (CI), as previously defined, was calculated for every IFFI class. The analysis was carried out separately for ascending and descending geometries and was applied to the entire set of interfering landslides, i.e. those located within a 100 m buffer around bridges.

**Fig. 4:**
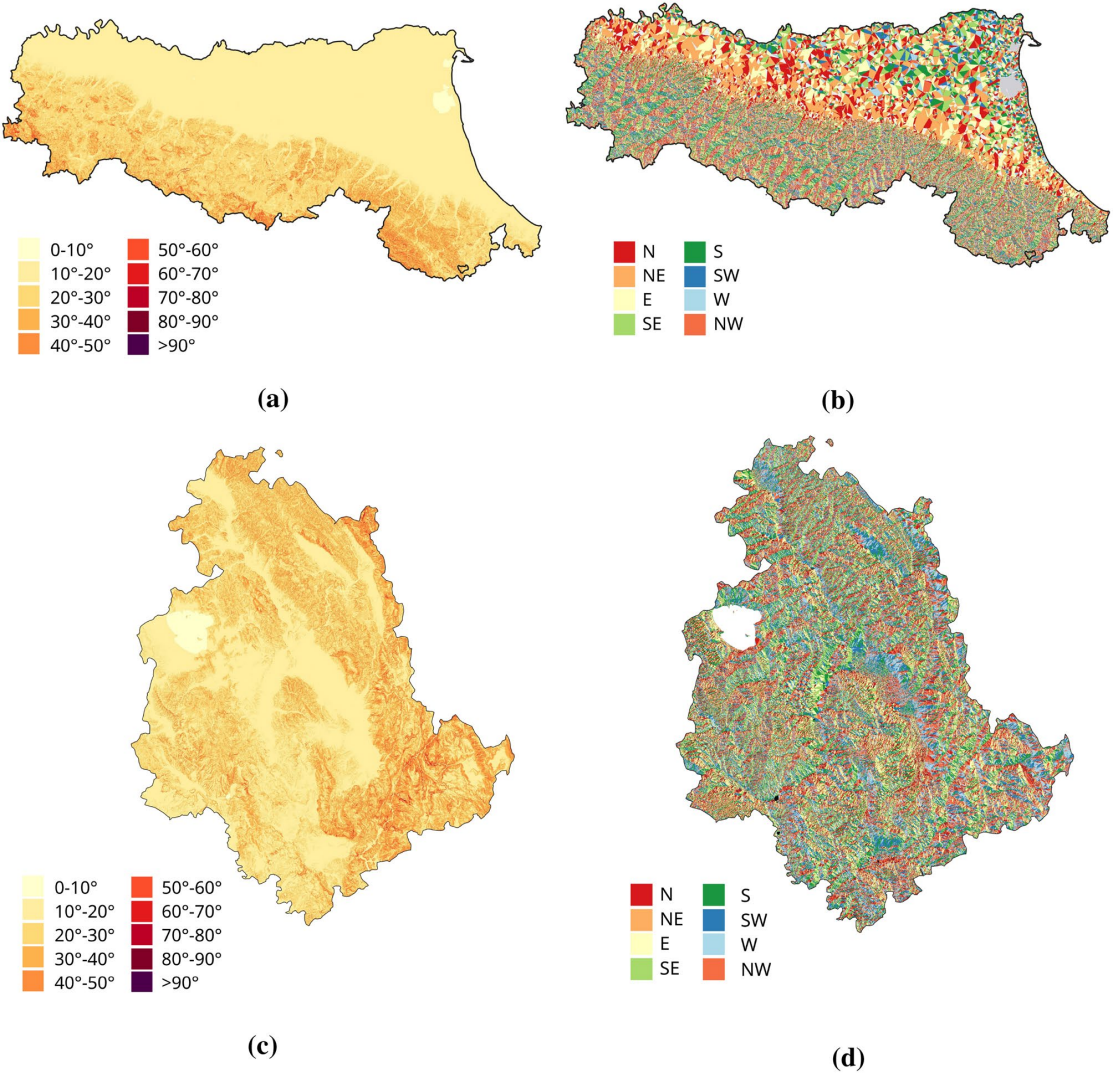
Geomorphological parameters for the Emilia-Romagna and Umbria regions: (**a,c**) slope and (**b,d**) aspect, derived from the 10 × 10 m TINITALY digital terrain model^48,49^ (https://tinitaly.pi.ingv.it/) and processed using QGIS v3.42.0 (https://qgis.org).

To complement this analysis, several geomorphological parameters were also considered, as they may affect radar visibility of the slopes regardless of the landslide mechanism. In particular, a slope-based analysis was performed focusing on two key variables: slope and aspect. Both parameters were derived from the TINITALY digital terrain model, with a spatial resolution of 10×10 m^48,49^, through processing in a GIS environment. These parameters are crucial in determining how slope surfaces are oriented with respect to the LOS of the SAR sensor, influencing the occurrence of geometric distortions such as foreshortening, layover and shadowing. Such distortions can significantly reduce the observability of certain portions of the landslide surface in interferometric data. Slope, defined as the inclination of the terrain with respect to the horizontal and expressed in degrees, was classified into ten intervals (from 0$^\circ$ to 90$^\circ$), to investigate how slope steepness affects the distribution of PSs (Fig. 4a,c). Aspect was instead categorised into eight cardinal sectors, considering that it is a circular variable (0$^\circ$–360$^\circ$) describing the orientation of the slope angle, that is, the direction in which the ground slope faces^50^ (Fig. 4b,d). Each sector was defined as a 45$^\circ$ interval centred on the corresponding cardinal direction, to ensure a consistent and comparable subdivision of slope exposure. For each interfering landslide, an average value of slope and aspect was determined by computing the mean of the geomorphological values within each polygon, extracted from the digital terrain model. These average values were then reclassified into the predefined classes and used to analyse, separately for each acquisition geometry, the influence of geomorphological factors on interferometric coverage.

### Analysis of deformation velocity and internal variability

**Fig. 5:**
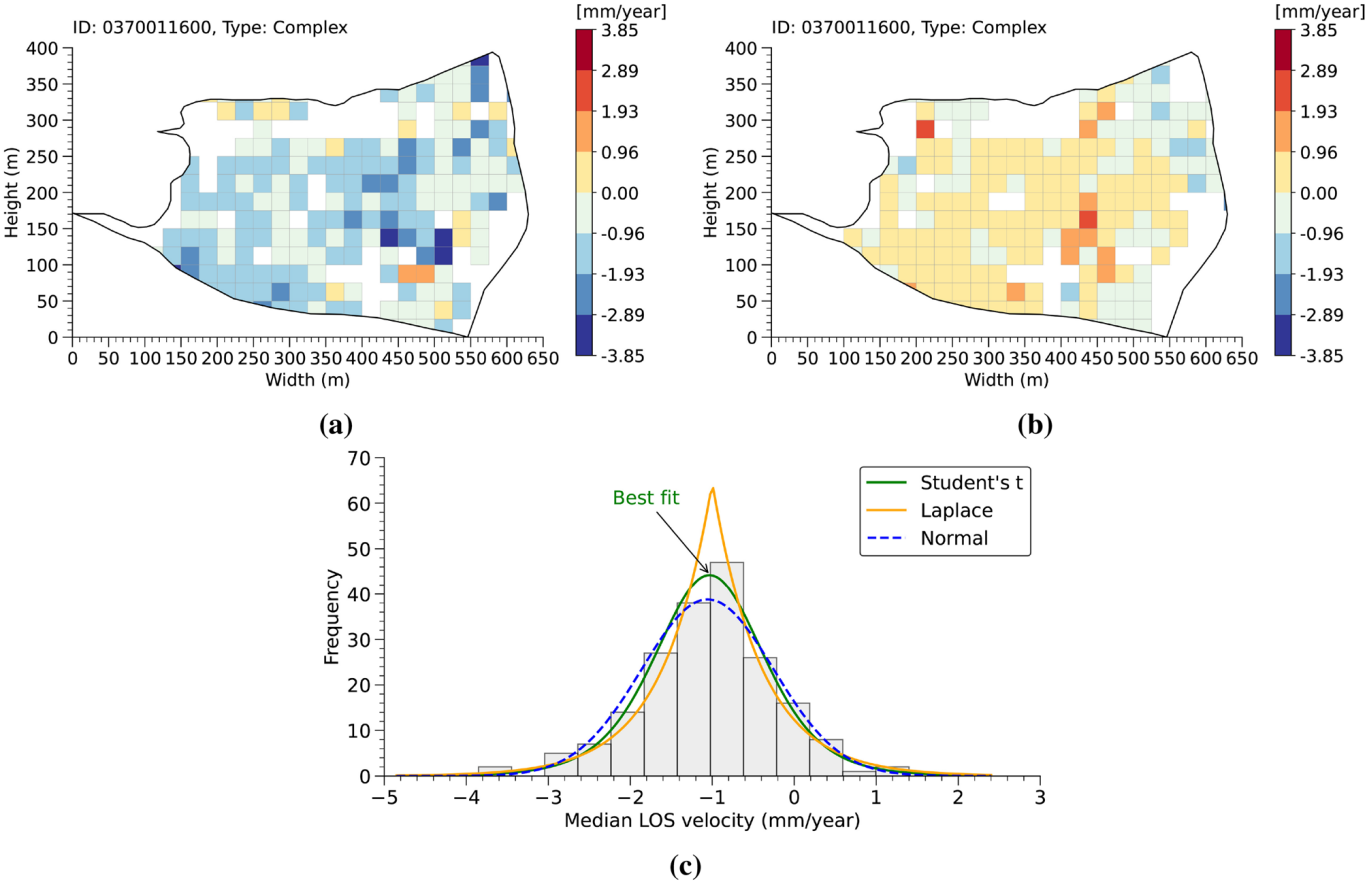
Example of the discretisation of landslide polygon ID 0370011600 (complex type) into 25×25 m square cells, with each cell assigned a representative value corresponding to the median LOS velocity of the PS contained within it. The results are shown for the ascending (**a**) and descending (**b**) geometries. Plot (**c**) shows the histogram of cell-based median LOS velocities for the same landslide (ascending geometry), illustrating the empirical deformation distribution fitted with three theoretical probability density functions (normal, Laplace, and Student’s t). The Student’s t distribution (green curve) provides the best fit to the observed data.

An analysis of deformation velocities along the LOS was performed to assess the capability of radar interferometry to detect the activity of interfering landslide phenomena and to describe the spatial distribution of deformation within each landslide polygon. The analysis is based on the processing of PS data, following an approach inspired by Crippa et al. (2021)^51^ but adapted to suit the scale, objectives, and characteristics of the available data. Specific methodological choices were introduced concerning the spatial discretisation, the descriptive statistics adopted, and the criteria used to classify the internal variability of the deformation. The analysis was limited to landslide polygons characterised by at least moderate interferometric coverage, to ensure the statistical reliability of the results. The two orbital geometries were processed separately, to preserve the directional differences related to the orientation of the phenomena with respect to the satellite LOS. Based on the previously introduced spatial grid, each landslide polygon was discretised using square cells of 25×25 m. Each cell was assigned a representative value corresponding to the median of the LOS velocities of the PSs contained within it (Fig. 5a, b). The use of the median provides a robust estimate of the local central tendency, reducing the influence of outliers or typical InSAR-related disturbances, such as atmospheric noise, anomalous reflections, or phase discontinuities. Once median velocity values were assigned to each cell, it became possible to describe the internal deformation pattern of each landslide polygon through a histogram of the cell-based median velocities, providing an empirical representation of the spatial variability of the phenomenon. To enable objective comparisons between different landslides and to overcome the limitations associated with the arbitrary choice of histogram bins, the observed distribution was subsequently approximated by a continuous theoretical function. Three candidate distributions (normal, Laplace, and Student’s t) were considered and, for each polygon, the best fitting model was selected based on the Kolmogorov–Smirnov (K–S) and Cramér–von Mises (CvM) tests (Fig. 5c). Polygons for which at least one distribution passed both statistical tests were retained for further analysis, whereas those not compatible with a reliable theoretical model were excluded. For each validated landslide, the first ($Q_1$) and third quartiles ($Q_3$) of the selected distribution were then calculated, representing the velocities below which 25$\%$ and 75$\%$ of the cells fall, respectively. The difference between these two values defines the interquartile range (IQR), which corresponds to the central portion of the distribution, containing 50$\%$ of the representative cell velocities.

To enable the comparison of internal heterogeneity across landslides characterised by different deformation levels, a dimensionless indicator was introduced, defined as:

1 $$\begin{aligned} {Q_{dev}} =\frac{Q_3 - Q_1}{Q_3 + Q_1} \end{aligned}$$

This index provides a concise measure of how deformation is spatially distributed or concentrated within an individual landslide phenomenon. By comparing the width of the interquartile range with the overall deformation level, the index offers an intuitive representation of internal heterogeneity. This formulation is normalised with respect to the intensity of the phenomenon, making it suitable for comparing landslides with different absolute velocity values. To establish operational thresholds for classifying internal variability, an empirical approach was adopted, based on both numerical and visual comparison between $Q_{dev}$ values and the shape of the associated theoretical distributions. Polygons with $Q_{dev}$ values $\ge$ 0.40 were found to exhibit marked internal heterogeneity, indicating significant spatial variability in deformation even when overall velocities were low. For instance, a case with $Q_1$ = 0.6 mm/year and $Q_3$ = 1.3 mm/year (yielding $Q_{dev}$ $\approx$ 0.4) clearly illustrates a heterogeneous deformation pattern. Values below 0.20 correspond to uniformly deforming phenomena, while intermediate values (0.20–0.40) identify cases with moderate variability. In general, the higher the $Q_{dev}$ value, the greater the heterogeneity in deformation across different portions of the landslide.

To assess internal heterogeneity, the analysis made it possible to classify the activity status of each landslide based on the theoretical modal velocity ($V_{PM}$), defined as the value corresponding to the peak of the probability density function of the theoretical distribution that best fits the observed data. This value represents the modal LOS velocity, i.e. the most probable deformation velocity within the landslide polygon. It serves as a synthetic and robust indicator of the overall deformational trend, being less sensitive to outliers than the mean and more representative than the median in cases of skewed distributions. The $V_{PM}$ value was then compared with a threshold of ±2 mm/year, which is commonly adopted to distinguish between interferometric signals compatible with noise and actual detectable ground deformations. This threshold is based on values reported in several studies^51–53^, and reflects the typical uncertainty associated with atmospheric effects, coregistration errors, and instrumental limitations. According to this criterion, absolute values of the modal velocity ($V_{PM}$) exceeding the 2 mm/year threshold were interpreted as indicative of significant deformation activity (active landslide), whereas values equal to or below this threshold were associated with a static condition.

A similar analysis was carried out for the interfering bridges, identified through a spatial buffer. For these structures, a 25×25 m grid was also applied, and the median LOS velocity of the PS within each cell was calculated. However, due to the limited number of PS available in the individual bridge cells, it was not possible to fit a statistically reliable distribution. Instead, the mode of the cell medians was adopted, providing a stable estimate consistent with the concept of the most representative velocity (analogous to $V_{PM}$ for landslides). This choice enables a direct comparison between each bridge and the associated landslide. The results of this comparison, discussed in the following sections, aim to assess whether the bridge is moving faster or slower than the interfering landslide, and to explore the potential structural and geotechnical implications.

## Results

### Filtered bridge–landslide sample

The spatial filtering carried out in the methodology phase, based on the definition of a buffer around the bridges, resulted in a substantial reduction in the number of landslides and infrastructures to be considered in the subsequent analyses. In Emilia-Romagna, only about 5$\%$ of the recorded landslides were found to be located in the proximity of a bridge, while in Umbria the percentage dropped to 1.5$\%$. Similarly, the number of infrastructures potentially subject to interaction was significantly reduced, to 2555 bridges in Emilia-Romagna and 448 in Umbria. The outcome of this spatial selection provides an updated overview of landslides and potentially interacting infrastructure in the two regions under investigation, and constitutes the reference framework for the following stages of the analysis.

### Impact of landslide type on SAR coverage

**Fig. 6:**
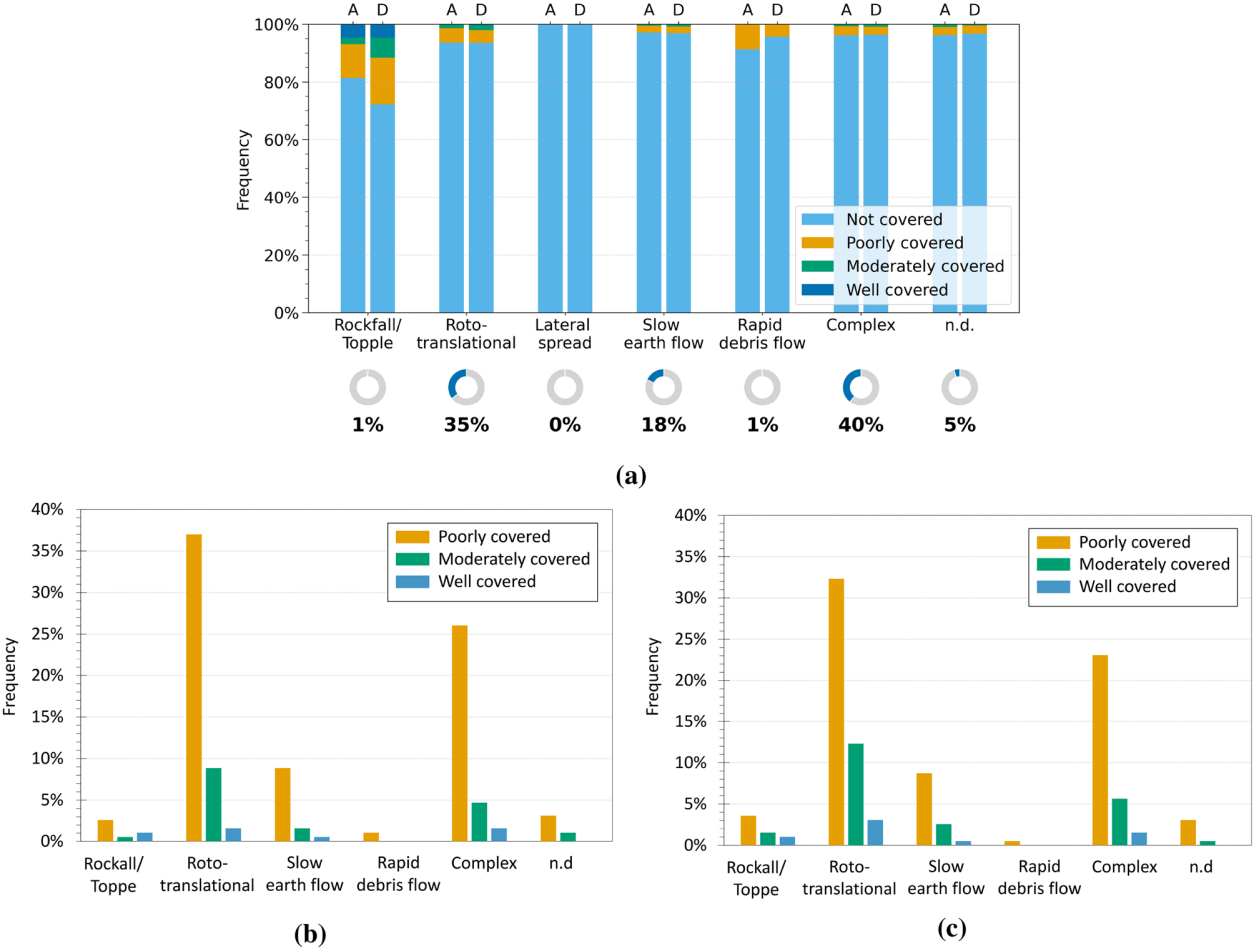
Analysis of InSAR coverage levels by landslide type in the Emilia-Romagna region. (**a**) Normalised 100$\%$ bar chart showing the distribution of coverage levels (CI) for each landslide type in ascending (A) and descending (D) geometries. The indicators below show the proportion of each type relative to all analysed landslides. (**b,c**) Charts for covered landslides only, in ascending (**b**) and descending (**c**) geometries, with percentages relative to the total number of covered landslides.

The analysis of how landslide type influences InSAR observability was first applied to the Emilia-Romagna region. As described in “Datasets and method”, each landslide was assigned an InSAR coverage level (CI), and the results were grouped according to the landslide type reported in the IFFI inventory. For each class, the percentage of events falling into the four coverage categories was calculated separately for ascending (A) and descending (D) geometries. The results were presented using 100$\%$ stacked bar charts (Fig. 6a), allowing a direct comparison of the distribution of coverage levels within each landslide type. The results show that, across all considered landslide types, the proportion of not covered events is generally high, suggesting a limited interferometric observability for a large part of the dataset. This trend can be attributed to factors such as vegetation cover, complex slope morphology, or the nature of the landslide itself, all of which may negatively affect the coherence of the radar signal.

To further investigate the landslides actually detected by InSAR data, the analysis was restricted to events falling within the three covered categories (poorly, moderately, and well covered) (Fig. 6b,c). For each type of landslide, the relative percentage with respect to the total number of covered landslides was calculated. This approach allows for the identification of the typologies that are most effectively represented within the informative subset of the interferometric dataset. The results show that roto-translational slides represent the most common landslide type among the covered events, in both geometries (Fig. 6b,c). Specifically, they account for approximately 37$\%$ (ascending) and 32$\%$ (descending) of the poorly covered class, with lower percentages in the moderately and well-covered categories. Complex landslides also occur with a significant frequency, representing 26$\%$ (ascending) and 23$\%$ (descending) of the poorly covered group, with progressively lower values in the remaining coverage classes. Slow earth flows are also relatively widespread, with a predominance in the poorly and moderately covered categories, whereas faster or more discontinuous types, such as falls/topples and rapid debris flows, are scarcely represented. These trends are consistent with findings in the literature that highlight that satellite interferometry is particularly well suited to monitoring slow-moving landslides, such as translational and rotational slides^54,55^, complex landslides^56,57^, and slow earth flows^58,59^. Conversely, interferometric observation generally proves more challenging in the case of rapid or discontinuous phenomena, such as rockfalls, topples, and debris flows, due to their high velocity and the lack of temporally coherent radar signals^17,31^.

**Fig. 7:**
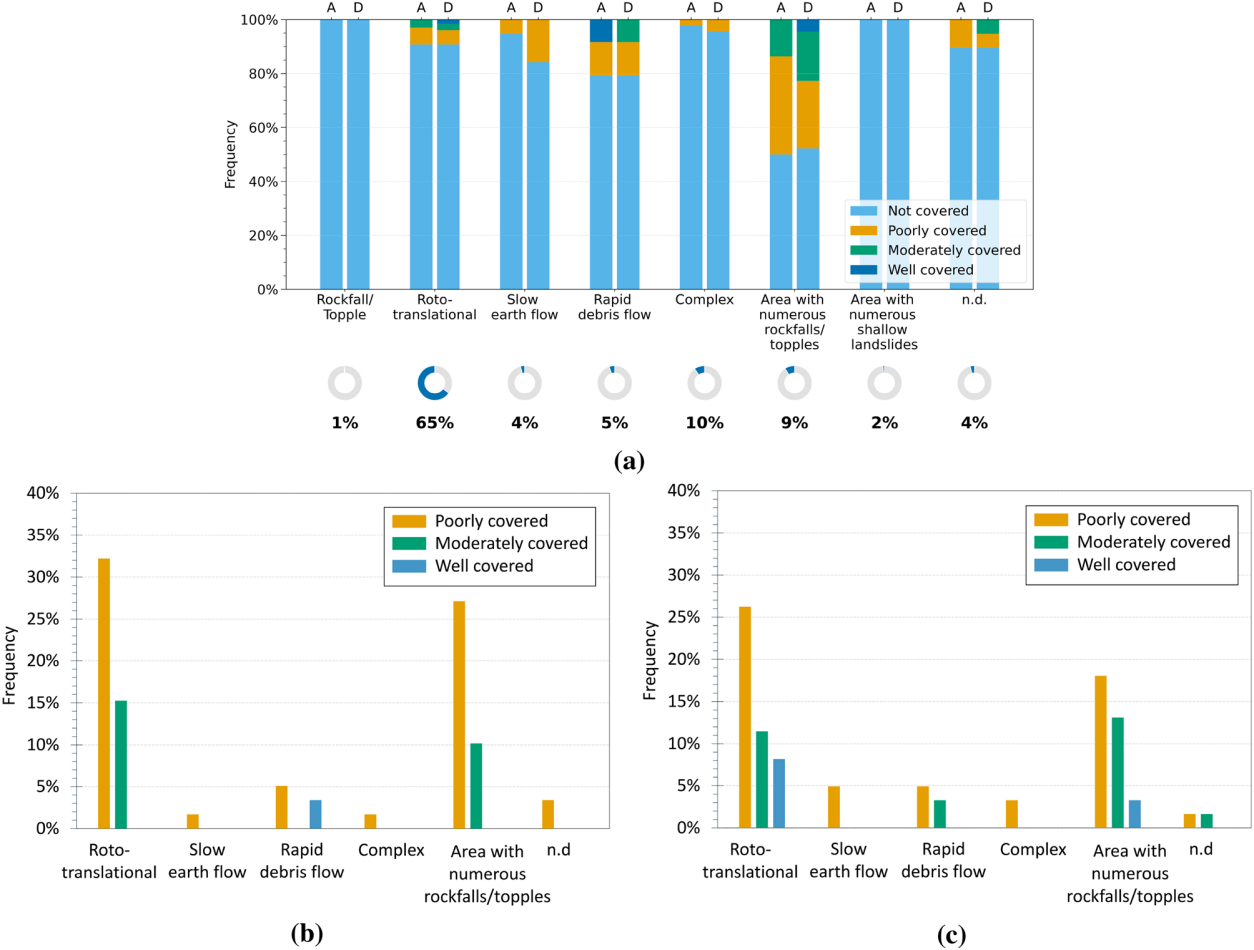
Analysis of InSAR coverage levels by landslide type in the Umbria region. (**a**) Normalised 100$\%$ bar chart showing the distribution of coverage levels (CI) for each landslide type in ascending (A) and descending (D) geometries. The indicators below show the proportion of each type relative to all analysed landslides. (**b,c**) Charts for covered landslides only, in ascending (**b**) and descending (**c**) geometries, with percentages relative to the total number of covered landslides.

The analysis was also extended to the Umbria region. Figure 7a shows the percentage distribution of InSAR coverage for each landslide type, distinguishing between ascending and descending geometries. Once again, the not covered category largely prevails, confirming the challenges of radar observation in many contexts. Figures 7b and c include only landslides that fall within the covered categories, allowing the most represented types to be identified within the informative subset. Roto-translational slides are the most frequent, representing about 32$\%$ (ascending) and 26$\%$ (descending) of the poorly covered class, with significantly lower percentages in the moderately covered class. Notably, no landslides of this type were classified as well covered in the ascending geometry. This absence is plausibly attributable to an unfavourable slope exposure with respect to the ascending LOS, which likely reduces the density of PSs compared to the descending geometry.

Compared to Emilia-Romagna, Umbria shows a different distribution of landslide types; for instance, complex landslides are scarcely represented. In contrast, areas affected by rockfalls/topples appear with notable frequency. Although such phenomena are generally associated with rapid and heterogeneous dynamics, their classification as “areas” and their occurrence on wide slopes may, in some cases, enhance their detectability through interferometric analysis. This is particularly true when the area includes stable or semi-stable portions (such as rock walls, infrastructures, or outcrops) that are able to retain sufficient coherence over time. In such settings, it is not the individual localised landslide that is effectively detected, but rather the cumulative deformational effects spread across the area. Despite the spatial heterogeneity of the signal, this can result in a meaningful level of radar coverage.

### Impact of geomorphological parameters on SAR coverage

**Fig. 8:**
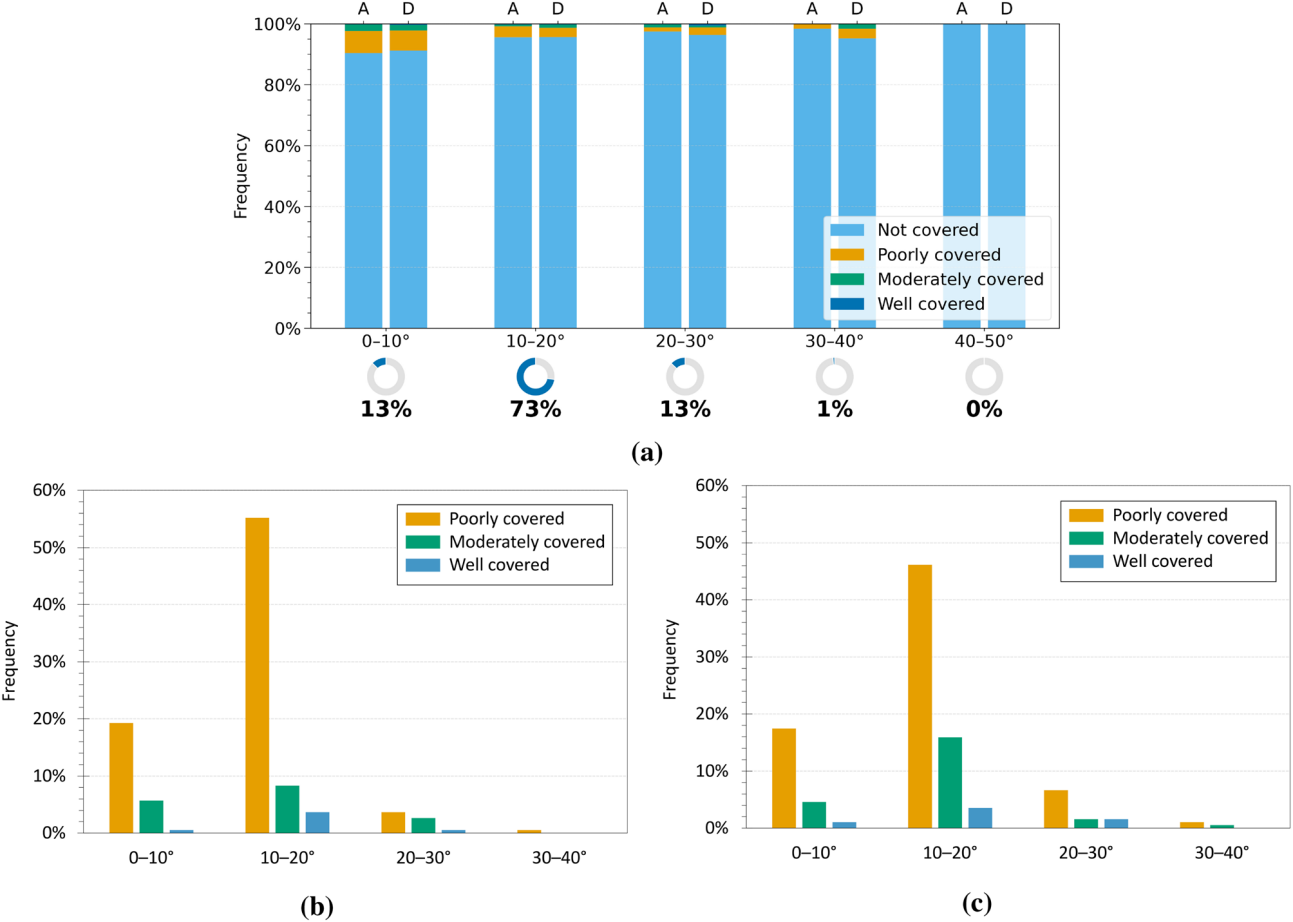
Analysis of InSAR coverage levels by slope class in the Emilia-Romagna region. (**a**) Normalised 100$\%$ bar chart showing the distribution of coverage levels (CI) for each slope class in ascending (A) and descending (D) geometries. The indicators below show the proportion of landslides in each class relative to the full dataset. (**b,c**) Charts for covered landslides only, in ascending (**b**) and descending (**c)**geometries, with percentages relative to the total number of covered landslides.

**Fig. 9:**
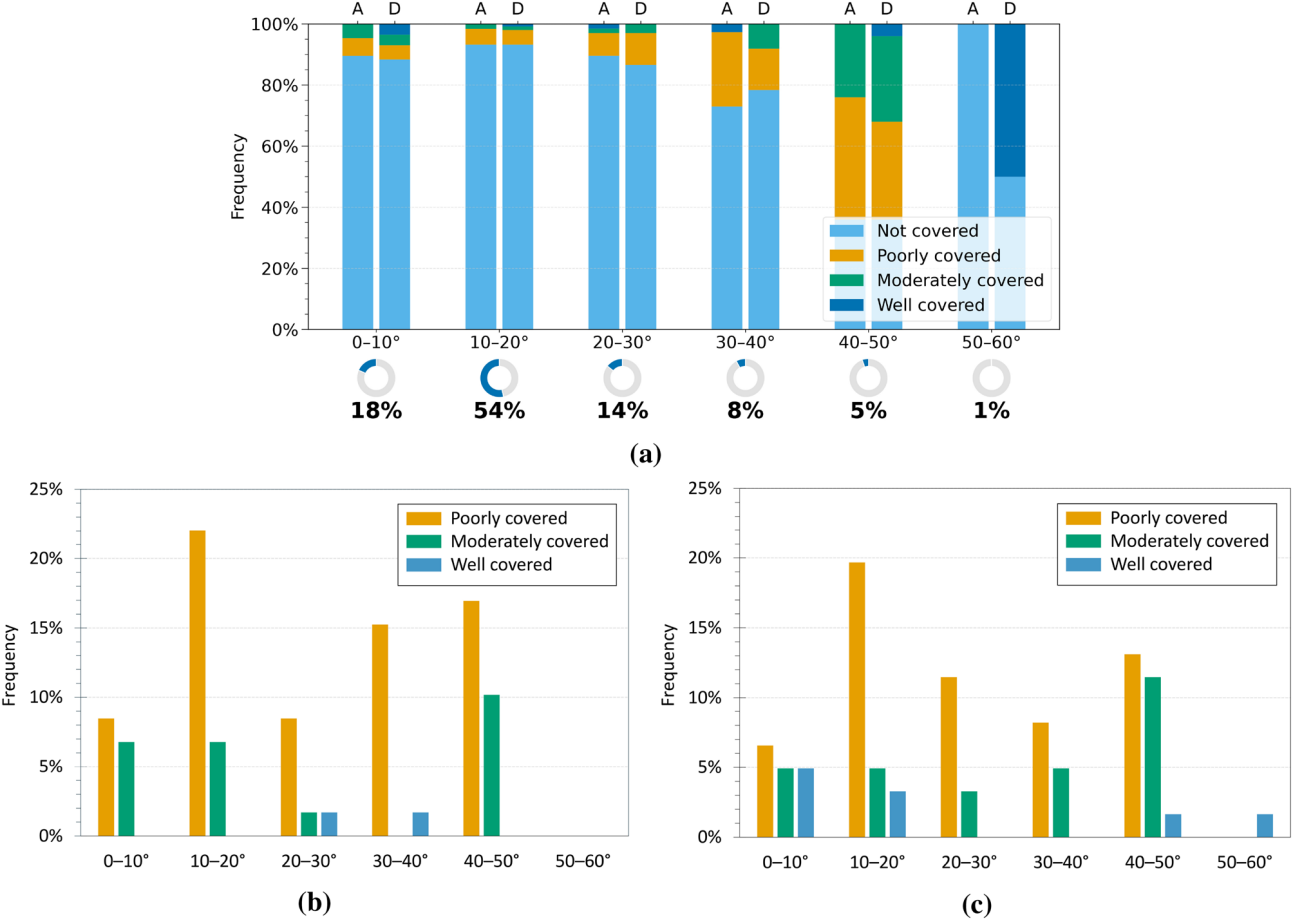
Analysis of InSAR coverage levels by slope class in the Umbria region. (**a**) Normalised 100$\%$ bar chart showing the distribution of coverage levels (CI) for each slope class in ascending (A) and descending (D) geometries. The indicators below show the proportion of landslides in each class relative to the full dataset. (**b,c**) Charts for covered landslides only, in ascending (**b**) and descending (**c**) geometries, with percentages relative to the total number of covered landslides.

The analysis was subsequently extended to investigate the influence of geomorphological parameters, specifically slope and aspect, on the detectability of landslides using InSAR data. Initially, the relationship between interferometric coverage and slope was examined by grouping interacting landslides into slope classes, based on the average inclination calculated for each polygon of landslide, as described in “Datasets and method”. Figures 8a and 9a show the distribution of InSAR coverage levels across the different slope classes, while Figures 8b,c and 9b,c present the distribution of only the landslides identified as covered, distinguishing between ascending and descending acquisition geometries. In both regions, most landslides fall into the “not covered” category, confirming the detection limitations observed earlier.

For Emilia-Romagna, the geometry-specific graphs (Fig. 8b,c) show a higher concentration of covered landslides on slopes with average inclinations between 10$^\circ$ and 20$^\circ$, which also represent the most populated class (73$\%$ of the dataset). Within this range, the largest proportion of well and moderately covered phenomena is observed in both ascending and descending geometries. Landslides with slopes between 0$^\circ$ and 10$^\circ$ (13$\%$ of the total) show lower coverage levels, while a sharp decrease is recorded for slopes steeper than 30$^\circ$. The 40$^\circ$–50$^\circ$ interval is completely devoid of detections, suggesting particularly unfavourable geometric conditions for radar observation. These findings suggest the existence of an optimal slope range (10$^\circ$–20$^\circ$) where radar visibility is enhanced. This can be attributed to a combination of factors: on the one hand, moderate slopes are often associated with active but slow-moving landslides, whose deformation rates are compatible with satellite acquisition intervals; on the other hand, such inclinations provide more favourable conditions with respect to the sensor acquisition geometry, reducing the likelihood of geometric distortions that typically affect steep slopes. Conversely, very steep slopes (>30$^\circ$) tend to generate unfavourable geometric effects that often hinder the formation of PS, while gentle slopes (<10$^\circ$), although geometrically observable, often exhibit limited interferometric coverage. This suggests that radar detectability in these settings is not controlled exclusively by acquisition geometry, but may also be influenced by surface cover characteristics and land use.

For the Umbria region, the slope-specific charts (Fig. 9b, c) show a distribution of interferometric coverage that is partly consistent with the pattern observed in Emilia-Romagna, although some relevant differences can be noted. As in the previous case, landslides are predominantly concentrated in the 0$^\circ$–10$^\circ$ (18$\%$ of the total) and 10$^\circ$–20$^\circ$ (54$\%$ of the total) slope classes, which together account for over 72$\%$ of the dataset. However, while in Emilia-Romagna coverage was strongly concentrated within the 10$^\circ$–20$^\circ$ class, in Umbria a broader coverage distribution is observed, including higher slope classes. In particular, the 30$^\circ$–40$^\circ$ and 40$^\circ$–50$^\circ$ classes, although representing a smaller share of the dataset (8$\%$ and 5$\%$ respectively), include a notable number of poorly and moderately covered landslides, with percentages that are significant in relation to the total number of covered events. In the 50$^\circ$–60$^\circ$ class, only one covered landslide is recorded; however, the very limited number of cases in this interval severely reduces its statistical relevance. These results suggest that in the Umbrian context the relationship between slope inclination and InSAR observability appears less distinct than in Emilia-Romagna. Although moderate slopes remain generally more favourable, a clearly optimal range is not observed and coverage, although limited, also extends to steeper slopes. This greater variability may reflect local factors, such as the specific morphological characteristics of the slopes or the different distribution of landslide types across the various slope classes.

**Fig. 10:**
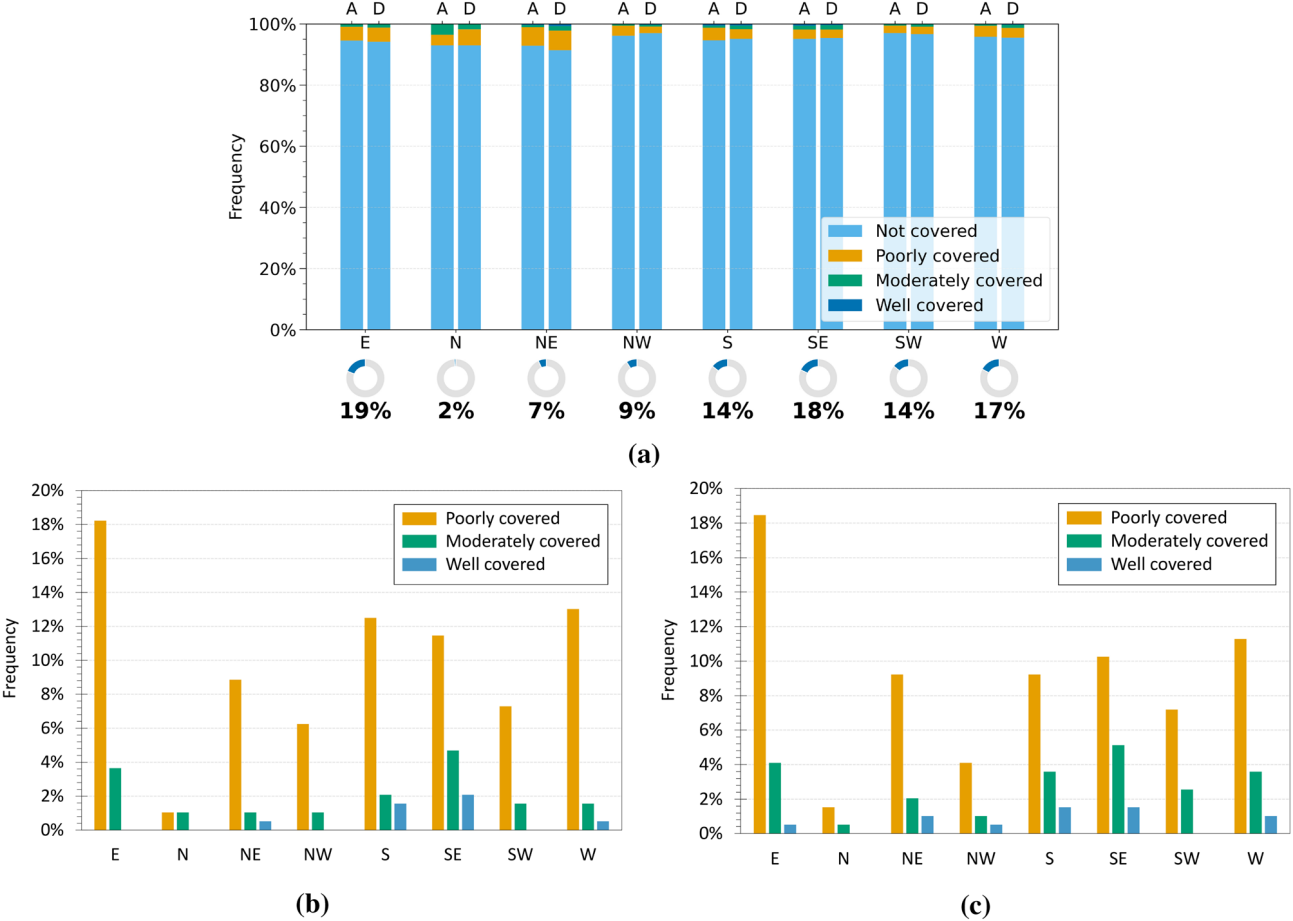
Analysis of InSAR coverage levels by aspect class in the Emilia-Romagna region. (**a**) Normalised 100$\%$ bar chart showing the distribution of coverage levels (CI) for each aspect class in ascending (A) and descending (D) geometries. The indicators below show the proportion of landslides in each class relative to the full dataset. (**b,c**) Charts for covered landslides only, in ascending (**b**) and descending (**c**) geometries, with percentages relative to the total number of covered landslides.

**Fig. 11:**
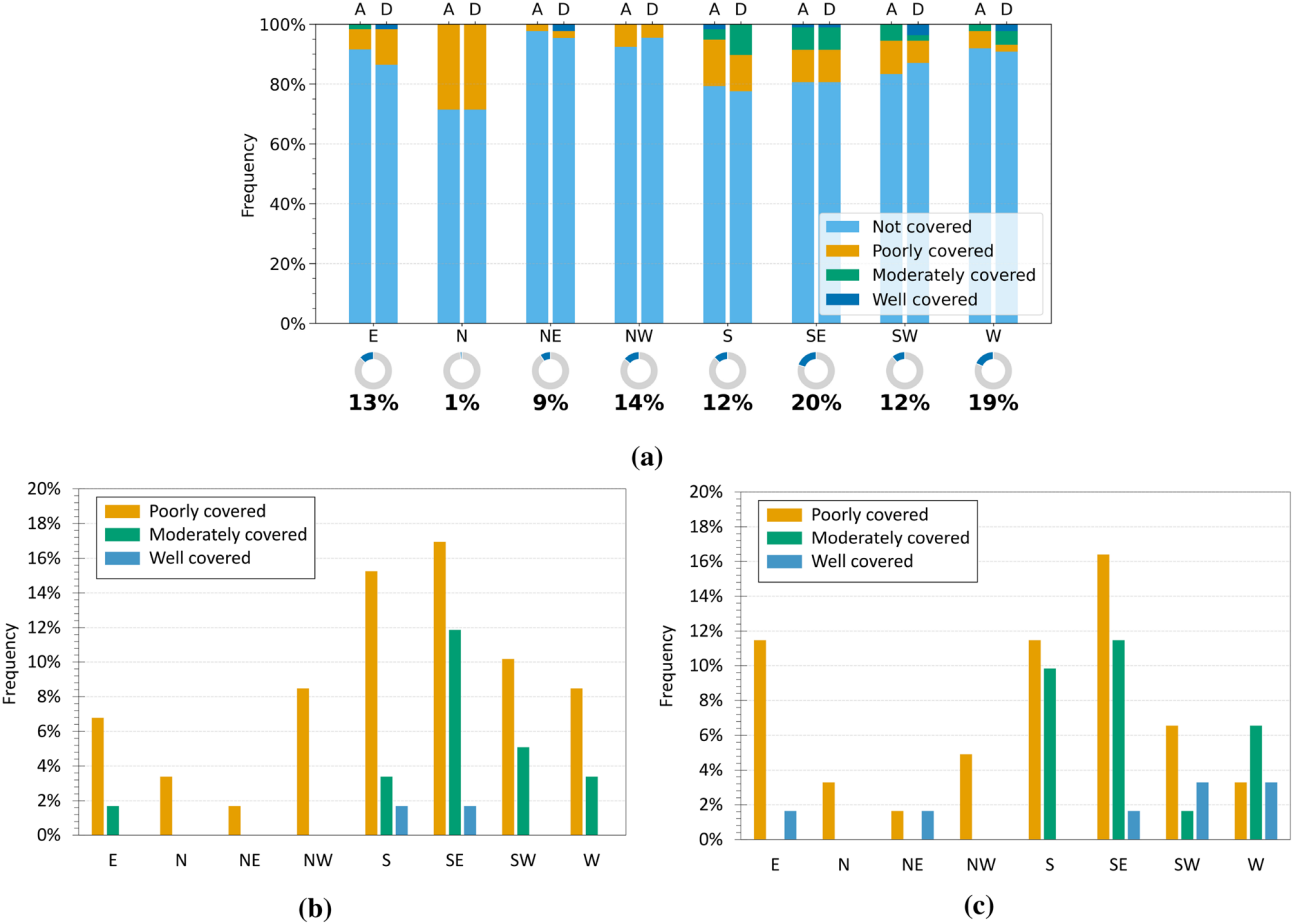
Analysis of InSAR coverage levels by aspect class in the Umbria region. (**a**) Normalised 100$\%$ bar chart showing the distribution of coverage levels (CI) for each aspect class in ascending (A) and descending (D) geometries. The indicators below show the proportion of landslides in each class relative to the full dataset. (**b,c**) Charts for covered landslides only, in ascending (**b**) and descending (**c**) geometries, with percentages relative to the total number of covered landslides.

Subsequently, attention was directed to the potential relationship between interferometric coverage (CI) and aspect, by analysing the results obtained for both regions. Figures 10a and 11a show the distribution of InSAR coverage levels across the different aspect classes, while Figures 10b, c and 11b, c present only the landslides that were actually detected, distinguishing between ascending and descending geometries. As also observed in the slope analysis, a predominance of landslides classified as not covered is evident across all aspect classes, confirming the general challenges of interferometric detection. In Emilia-Romagna, the analysis of the landslides that were effectively detected (Fig. 10b,c) reveals that a grater coverage occurs on slopes facing east, south, south-east, and west, with east-facing slopes showing the highest proportion of covered cases in both geometries. This trend may result from a combination of factors, including the actual distribution of landslides in different slope orientations and the geometric interaction between the aspect and the satellite LOS. However, it is important to note that the distribution of landslides among the aspect classes is uneven, as shown by the percentage indicators below the main graph: for instance, only 2$\%$ of the landslides face north, while 19$\%$ are located on east-facing slopes.

With regard to Umbria, the geometry-specific graphs (Fig. 11b,c) show improved coverage for slopes facing south-east, south, and south-west, whereas landslides oriented towards the north and north-east are mostly undetected. The behaviour is consistent across both ascending and descending geometries, with only slight differences in the percentage distribution. The poorly covered class remains the most frequent among the detected landslides across nearly all aspect directions. In Umbria, improved coverage is observed for south-east, south, and south-west exposures, whereas north- and north-east-facing slopes are rarely detected (Fig. 11b, c). The behaviour is similar across both orbital geometries, with only minor variations. The poorly covered class remains predominant among detected landslides across nearly all orientations. Once again, the distribution of landslides across the different aspect classes is uneven, as indicated by the percentages shown below the main chart: for instance, only 1$\%$ of the landslides are north-facing, whereas 20$\%$ are associated with south-east slopes. Overall, although some recurrent patterns emerge, especially improved visibility for south and south-east facing slopes, the influence of aspect on interferometric detectability appears weaker than that of slope or landslide type, and its effect is difficult to isolate due to the uneven distribution of landslides among orientation classes.

### Analysis of deformation velocity and internal spatial variability

As outlined in “Datasets and method”, the analysis of deformation velocities and internal variability was carried out exclusively on landslide polygons classified as well or moderately covered, to ensure a sufficient density of PSs for reliable processing. Based on this criterion, 29 landslides were selected in Emilia-Romagna and 15 in Umbria. However, only a subset of these proved suitable for the statistical analysis of the internal velocity distribution. In some cases, the limited spatial extent of the polygon resulted in an overly coarse discretisation, while in others, the low variability among recorded values prevented the construction of a meaningful histogram and its subsequent approximation by a continuous theoretical distribution. Although these conditions did not compromise the overall InSAR coverage quality, they did hinder a robust characterisation of deformation heterogeneity and led to the exclusion of certain landslides from further analysis.

For the Emilia-Romagna region, the analysis of the $Q_{dev}$ index revealed a marked prevalence of heterogeneous deformation behaviour. In the ascending geometry, 88$\%$ of the validated landslide polygons were classified as heterogeneous ($Q_{dev}$ $\ge$ 0.40), while the remaining 12$\%$ were identified as moderately heterogeneous (0.20 $\le$ $Q_{dev}$ < 0.40); no cases exhibited a homogeneous internal distribution. In the descending geometry, internal variability appeared more evenly distributed: 70$\%$ of the phenomena were classified as heterogeneous, 26$\%$ as moderately heterogeneous, and 4$\%$ as homogeneous. For the Umbria region, the results show a similar pattern in the ascending geometry, with 77$\%$ of the cases classified as heterogeneous, 8$\%$ as moderately heterogeneous, and 15$\%$ as homogeneous. In the descending geometry, a more balanced distribution is observed: 38$\%$ of the cases were classified as moderately heterogeneous, 31$\%$ as heterogeneous, and the remaining 31$\%$ as homogeneous. In general, the analysis shows that the internal deformation heterogeneity represents the predominant condition in most of the landslides examined. This outcome reflects the inherently complex and non-uniform nature of landslide phenomena, which may exhibit highly variable displacement patterns depending on multiple factors, including lithological heterogeneity, the geometry of the landslide body and local variations in hydrogeological conditions. Thus, interferometric analysis proves to be effective in highlighting such spatial variability, providing a more detailed picture of the internal deformation dynamics within a landslide.

Regarding the state of activity, most of the landslides analysed in both Emilia-Romagna and Umbria are classified in the IFFI inventory as dormant. A smaller proportion is identified as relict or falls under the category of active/reactivated/suspended. The latter refers to a potentially dynamic condition, though not necessarily ongoing. The IFFI classification is based on historical information, previous observations or archived reports, and may include both currently active phenomena and instabilities that were reactivated in the past or are currently inactive but still susceptible to future reactivation. The interferometric analysis generally confirmed the activity status reported in the inventory, indicating for most landslides a condition consistent with the IFFI classification. In particular, for those classified as active/reactivated/suspended, the results revealed a certain degree of variability: in some cases, the observed velocities exceeded the ±2 mm/year threshold, confirming the presence of active deformation during the EGMS monitoring period; in others, the velocities remained below this threshold, suggesting a currently static phase. These results, which are consistent with the nature of the IFFI classification, confirm the usefulness of satellite data as a complementary tool for dynamic updating of information on deformation activity.

**Fig. 12:**
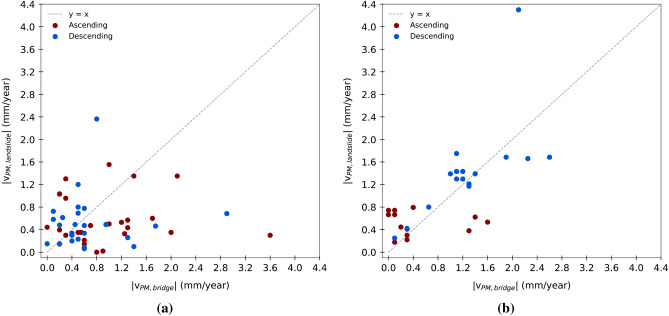
Relationship between the modal velocity ($V_{PM}$) recorded on bridges and that observed in the interfering landslide body for the Emilia-Romagna (**a**) and Umbria (**b**) region. Each point represents a bridge–landslide pair. Velocities are expressed in absolute values. The dashed line indicates the condition of equality (y=x).

In addition, to investigate the relationship between the deformation behaviour observed on the infrastructure and that recorded within the interacting landslide body, a direct comparison was carried out between the modal velocity ($V_{PM}$) values computed for each bridge and those corresponding to the associated landslide volume. The results are shown in Figures 12a,b, where each point represents a bridge–landslide pair, distinguished by orbital geometry. The dashed line represents the bisector y=x, indicating the condition in which the deformation velocity measured on the bridge is equal to that observed in the landslide. To ensure a consistent comparison, unaffected by the direction of movement along the LOS, positive for motion towards the satellite and negative for motion away, velocities are expressed in absolute value.

In the case of Emilia-Romagna, both in ascending and descending geometries (Fig. 12a), the data show a slight prevalence of points below the bisector. This indicates that, in several cases, the deformation velocities observed on the bridges are higher than those recorded within the corresponding landslide bodies. Although it might be expected that the primary deformational phenomenon (the landslide) would exhibit greater velocities than the structure it affects, this is not always the case. Various factors may influence the behaviour recorded on the bridges, including the geometric and mechanical characteristics of the structure, the type and depth of the foundations, the relative position with respect to the landslide body, and potential local effects unrelated to the landslide itself, such as differential settlements or independent movements.

In the case of Umbria (Fig. 12b), a partially different pattern emerges compared to Emilia-Romagna. In both orbital geometries (ascending and descending), the distribution of points with respect to the bisector reveals a clearer tendency towards higher deformation velocities within the landslide areas than on the associated structures. This trend is particularly pronounced in the descending geometry, where most data points lie above the bisector, indicating that interfering landslides generally exhibit higher modal velocities than the corresponding bridges. Nevertheless, even in this context, the relationship between the two velocities is neither linear nor consistent, but rather influenced by a range of local factors that contribute to more complex and variable deformational behaviour than might be expected.

## Discussion

The analysis carried out in Emilia-Romagna and Umbria regions focused on landslides interfering with bridges, identified through a 100 m buffer to isolate the most infrastructure-relevant cases and assess radar visibility as a function of geomorphological, geometric, and local land-cover factors. Processing ascending and descending geometries separately proved essential to highlight the influence of viewing direction and the complementarity between datasets: phenomena poorly visible in one geometry were often detectable in the other, confirming that integrating both perspectives reduces shadow zones and provides a more complete picture of interferometric observability.

From a typological perspective, slides are by far the most consistently observable category in both regions, while other types are less represented. In Emilia-Romagna, complex landslides and, to a lesser extent, slow earth flows are also present; in Umbria, the contribution of complex landslides is marginal, and inventory areas associated with diffuse rockfalls or topples appear. These do not correspond to single rapid events but to larger zones where coherent portions of the slope (rock faces, outcrops, or infrastructures) ensure sufficient radar signal. Such differences reflect the distinct lithological and morphological settings of the two regions but also confirm that slides remain the most reliably monitored landslide type using InSAR data.

Slope plays a major role in determining detectability. In both regions, slopes between 10$^\circ$ and 20$^\circ$ show the highest probability of detection, since they combine favourable LOS geometry with deformation rates compatible with the temporal resolution of the satellite. Very steep slopes (>30$^\circ$) are penalised by geometric effects such as layover and shadowing. Conversely, gentle slopes (<10$^\circ$), although geometrically observable, often exhibit limited interferometric coverage, suggesting that detectability in these settings is influenced by factors beyond acquisition geometry. In Umbria, detectable landslides also occur on steeper slopes, likely reflecting local morphological and kinematic variability.

Aspect exerts a relatively weaker influence, acting mainly as a secondary factor that modulates the density of coherent points according to orientation with respect to the LOS, but it cannot alone explain the presence or absence of radar coverage. It should therefore be interpreted together with other parameters for a more comprehensive understanding of interferometric observability. Beyond coverage, the analysis highlighted the significance of internal deformation variability. The $Q_{dev}$ index revealed that heterogeneous behaviour is prevalent, consistent with the intrinsic complexity of landslide bodies, while the modal velocity ($V_{PM}$) proved to be a robust and synthetic indicator of the general deformation state, confirming or refining inventory information within the ±2 mm/year threshold.

Finally, the comparison between velocities observed on bridges and those detected in interfering landslides showed that the relationship is not linear: in some cases bridges appear to move faster than the landslide, in others the opposite. This outcome should not be interpreted as direct evidence of structural instability, but rather as an indicator of local complexity, where several factors come into play, including the type and depth of the foundations, the position of the structure relative to the most active portions of the landslide, the nature of radar sampling on bridges, and possible non-landslide-related contributions (thermal deformation, local settlements, cyclic loads). The distribution of PS on bridges, often limited to exposed and sensitive elements, also contributes to this variability. In this sense, the observed discrepancies are primarily signals of complexity and cases that should be further investigated using complementary methods.

## Conclusion

The study provided a systematic assessment of the interferometric observability of landslides interacting with linear infrastructure, integrating EGMS data with the IFFI inventory to identify the conditions under which significant deformations can be detected. The analysis conducted in Emilia-Romagna and Umbria confirmed that the effectiveness of radar observation is strongly influenced by the geomorphological and geometric characteristics of the slopes, highlighting differences related to both morphology and the orientation of the phenomena. The separate analyses of ascending and descending geometries revealed their complementarity, suggesting that their integration represents a potential advancement toward a more comprehensive characterization of deformation phenomena. Slides proved to be the most consistently observable type, whereas complex landslides, flows, and falls were less detectable, reflecting the different responses of movement mechanisms to radar acquisition geometry. The slope range between 10$^\circ$ and 20$^\circ$ resulted to be the most favourable for detection, confirming the key role of slope morphology in determining radar visibility, while aspect, although modulating point density, did not prove to be a discriminant factor unless considered together with other parameters. The assessment of internal variability within well-covered landslides, using indicators such as modal velocity ($V_{PM}$) and the $Q_{dev}$ index, showed that heterogeneity is a recurring feature, consistent with the inherent complexity of landslide bodies. In several cases, interferometric results suggested more active behaviour than that reported in the inventory, pointing to the potential of satellite observations for targeted updates. The comparison between velocities measured on bridges and those detected in the interfering landslides revealed differences mainly related to local complexity rather than to actual structural instability. This finding highlights the need to integrate satellite data with traditional surveys and numerical modelling to distinguish deformations effectively induced by landslides from other independent contributions. Overall, the proposed method provides an operational framework for large-scale evaluation of landslide–infrastructure interaction, serving as a valuable preliminary support for risk management and the planning of more detailed investigations.

## Data Availability

The datasets analysed during the current study are publicly available. Interferometric data are provided by the European Ground Motion Service (EGMS) (https://egms.land.copernicus.eu/), while landslide inventory data are available from the Italian Landslide Inventory (IFFI) (https://idrogeo.isprambiente.it/).
